# Diffusion in crowded biological environments: applications of Brownian dynamics

**DOI:** 10.1186/2046-1682-4-3

**Published:** 2011-03-02

**Authors:** Maciej Długosz, Joanna Trylska

**Affiliations:** 1 Interdisciplinary Centre for Mathematical and Computational Modeling, University of Warsaw, Żwirki i Wigury 93, 02-089 Warsaw, Poland

## Abstract

Biochemical reactions in living systems occur in complex, heterogeneous media with total concentrations of macromolecules in the range of 50 - 400 $\frac{mg}{ml}$. Molecular species occupy a significant fraction of the immersing medium, up to 40% of volume. Such complex and volume-occupied environments are generally termed 'crowded' and/or 'confined'. In crowded conditions non-specific interactions between macromolecules may hinder diffusion - a major process determining metabolism, transport, and signaling. Also, the crowded media can alter, both qualitatively and quantitatively, the reactions in vivo in comparison with their in vitro counterparts. This review focuses on recent developments in particle-based Brownian dynamics algorithms, their applications to model diffusive transport in crowded systems, and their abilities to reproduce and predict the behavior of macromolecules under in vivo conditions.

## Introduction

Intracellular organelles are packed with small solutes, macromolecules, membranes and skeletal proteins. Such crowding is characteristic not only of the cell's interior but also for extracellular tissues [1]. The kinetics and thermodynamics of macromolecular processes and biochemical reactions taking place in vivo are known to be affected by complex, volume-occupied environments [2-5].

More and more experimental methods have become available to investigate macromolecules under in vivo conditions. These include nuclear magnetic resonance [6-8] and fluorescence spectroscopies [9]. Techniques such as SPT (single-particle tracking) [10,11], FRAP (fluorescence recovery after photobleaching) [12,13], FCS (fluorescence correlation spectroscopy) [14] have been applied to measure diffusion constants of macromolecules in the cytoplasm and membranes. All experiments show that diffusion of proteins in vivo is significantly reduced compared to dilute conditions. In the cytoplasm of eukaryotic cells, diffusion of both large and small molecules is slowed down three to four times [1]. FRAP measurements of the diffusion coefficient of GFP in the *Escherichia coli *(*E. coli*) cytoplasm [15,16] yield about 10 times smaller values than at infinite dilution in water.

Theories of diffusion for colloidal soft matter are well established. Within their framework, the behavior of colloidal systems (both dilute and concentrated), consisting of mesoscopically large colloidal particles dispersed in low-molecular-weight solvent, can be simulated, reproduced, and more importantly predicted [17-21]. In cell biology, however, an appropriate theory of diffusion phenomena is still lacking. Complications arise due to the cellular heterogeneity. Moreover, there are different cell types whose compositions vary over space and time on the scales associated with diffusive motion and the knowledge of the surroundings of diffusing species is essential for successful applications of theoretical approaches. Unfortunately, even for the best-studied systems, experimental data is too scarce to describe elementary processes involving macromolecules, their timing and spatial distribution.

Simulation methods used to study the in vivo diffusion of macromolecules should take into account their biological localization, sizes and shapes, and positions in three dimensional space. Intermolecular interactions (both specific and non-specific, repulsive and attractive) should be also included [22]. Ideally, the modeling should be performed at atomic resolution. However, due to the *μ*m size of an average cell and a large number of components occupying its volume, reductionist (coarse-grained) approaches are unavoidable. The simulation methods [22] that can be applied to directly account for the crowded conditions, sizes and shapes of molecules and their interactions, as well as other features of the cellular environment, include different variants of particle-based Brownian dynamics (BD) [23-26]. In this review, we describe recent applications of BD to study diffusion in the crowded, in-vivo-like systems. Examples of different modeling approaches, either coarse-grained or more elaborate, are presented and discussed.

## Diffusion in dilute and crowded solutions

In dilute solutions, diffusional properties of molecules treated as rigid bodies are determined by their sizes, shapes, temperature, and solvent viscosity. The complete information required to describe translational and rotational motions of Brownian particles in dilute solutions is contained in their diffusion tensors. Single-particle diffusion tensor, *D*, is represented with a symmetric, 6×6 matrix containing 3×3 blocks related to the translational (*tt*) and rotational diffusivities (*rr*) and their couplings (*rt, tr*) [26]:

(1) $$D=\left(\begin{array}{cc} D^{tt} & D^{tr}\\ D^{rt} & D^{rr} \end{array}\right)$$

The diffusion tensors of molecules can be obtained from rigid-body hydrodynamic calculations [27]. Translational diffusion coefficient is an average over the diagonal terms of *D^tt^*. The rotational dynamics of a free diffusing molecule can be predicted from the eigenvalues of *D^rr ^*[28]. For a spherical molecule, diffusion is isotropic, the diffusion tensor is diagonal, and the molecule can be described with a single translational and a single rotational diffusion coefficient. Translational diffusion coefficient (*D^trans^*) determines the time dependence of the diffusion of the particle's center of mass. The mean square displacement (*MSD*) of a freely diffusing (in three dimensions) particle's center of mass changes linearly with time *t *according to the following equation [29]:

(2) $$MSD(t)=6\cdot D^{trans}\cdot t$$

with the *D^trans ^*as a proportionality constant.

In biological setup, solutes interact with their environments and other molecules which affects their Brownian motion, thus single-particle diffusion tensors are no longer the unique determinants of diffusion. Nevertheless, the relation between the *MSD *and time *t *(not necessarily linear) can still be used to measure the apparent translational diffusion of molecules [4,11].

## Particle-based Brownian dynamics algorithms

Brownian dynamics is a stochastic simulation approach with continuum space and time. In BD the molecules exhibit noise as they are propagated according to the Langevin equation [30]. The equation contains random and frictional forces that are intended to represent the interaction between the diffusing molecules and solvent (treated implicitly). High-friction limit is assumed; momentum relaxation is much faster than position relaxation, thus the equations of motions do not contain particles' velocities. Below, we briefly describe two common BD algorithms.

### Brownian dynamics of rigid, arbitrarily shaped objects

The propagation scheme for a number of rigid bodies, described either at atomic level or with coarse-grained models, can be written as [23,26]:

(3) $${\vec{x}}_{i}={\vec{x}}_{i}^{o}+\frac{\Delta t}{k_{B}T}D_{i}{\vec{M}}_{i}+{\vec{R}}_{i}\left(\Delta t\right)$$

where *i *runs over molecules, Δ*t *is the time step, and $\vec{x}$ is the vector describing the position of the center of diffusion ($\vec{r}$) and orientation ($\vec{\phi }$) of the *i*-th molecule, $\vec{x}={\left(\vec{r},\vec{\phi }\right)}^{T}$. $\vec{M}$ is a generalized force acting on a given particle having two components: the force acting on the diffusion center ($\vec{F}$) and the torque $\left(\vec{T}\right),\vec{M}={\left(\vec{F},\vec{T}\right)}^{T}$. $\vec{R}$ (Δ*t*) is a random displacement vector resulting from the Brownian noise, with zero mean and the variance-covariance given with:

(4) $$<{\vec{R}}_{i}\left(\Delta t\right){\vec{R}}_{j}\left(\Delta t\right)>=2D_{i}\delta_{ij}\Delta t$$

Here, *D *is the precomputed and constant in the simulation 6×6 diffusion tensor of the *i*-th molecule at its diffusion center. Random displacements of particles can be computed via Cholesky decomposition of *D *[23]. Intermolecular hydrodynamic interactions between diffusing bodies are neglected.

A simplified version of the above algorithm [25], which assumes isotropic properties of molecules and replaces their diffusion tensors with translational and rotational diffusion coefficients, has been widely used. Apart from treating the diffusing molecules as spheres, this simplification also ignores the coupling between translational and rotational diffusion. It is however questionable, whether using pre-averaged diffusion tensors is justified for nonglobular molecules (e. g., see the discussion on the influence of anisotropy on transport and association rates of model molecules [31] or on the diffusion of an ellipsoid near planar surfaces [27]).

### Brownian dynamics with hydrodynamic interactions

When hydrodynamic interactions (HI) are considered in BD simulations, the diffusion tensor of the entire system (6N×6N, with N being the number of spherical molecules or the number of spherical pseudo-atoms in case of more elaborate coarse-grained molecular models [32]) is used to propagate particles. This tensor depends on the system's configuration and varies with time. The propagation scheme can be written as [23,24]:

(5) $$\vec{x}={\vec{x}}^{o}+\left(\nabla D\right)\Delta t+\frac{\Delta t}{k_{B}T}D\vec{M}+\vec{R}\left(\Delta t\right)$$

When the Rotne-Prager-Yamakwa [33,34] form of the diffusion tensor is used (see below) the gradient term in the above equation (∇*D*) vanishes. The 6N-dimensional random displacement vector has zero mean and fulfills <$\vec{R}$ (Δ*t*)$\vec{R}$ (Δ*t*) > = 2*D*Δ*t*. Again, random displacements of particles can be computed via the Cholesky decomposition of *D *[23]. However, the decomposition of *D *must be performed at each BD step due to its dependence on the positions of particles.

### Intermolecular interactions

The influence of environments on the diffusion of macromolecules manifests through intermolecular forces acting on individual particles. The types and functional forms of interactions included in BD simulations vary depending on the level of detail used to describe the studied systems. Different types of intermolecular interactions that can be modeled in BD simulations are briefly described below.

### Electrostatics

When diffusing molecules are described in a reduced way with spherical subunits (e. g., as in case of model spherical proteins [35-37] or bead-models of flexible polymers [38]), the DLVO (Derjaguin - Landau - Verwey - Overbeek) approach (or the Debye-Hückel approximation), commonly applied in the studies of colloid suspensions, can be used [39]. Spherical molecules (of radii *R_i_*, *R_j_*) with central net charges (*Q_i_*, *Q_j_*) interact via pairwise additive potentials:

(6) $$V\left(r_{ij}\right)=\frac{Q_{i}Q_{j}}{4\pi \epsilon_{o}\epsilon \left(1+kR_{i}\right)\left(1+kR_{j}\right)r_{ij}}e^{-\kappa (r_{ij}-R_{i}-R_{j})}$$

Where ϵ*_o _*is the vacuum permittivity, ϵ is the dielectric constant of the immersing medium, *κ *is the inverse of the Debye screening length, and *r_ij _*is the separation between molecules. However, the above description is not valid for highly charged systems such as nucleic acids where the linearized form of the Poisson-Boltzmann equation [40] is not applicable and there is strong electrostatic coupling between the molecular and ionic species. In such cases, the net charges can be replaced with renormalized charges [41].

A more sophisticated approach to treat electrostatic interactions in BD simulations of rigid molecules with atomic detail [42,43] is based on the model of effective charges developed by Gabdoulline and Wade [44]. In this approach, each molecule is described with a limited set of optimized, discrete, effective charges immersed in an uniform dielectric. These effective charges are derived by fitting the electrostatic potential resulting from the Debye-Hückel approximation to the external molecular potential obtained from the numerical solution of the Poisson-Boltzmann equation. Electrostatic energies (and thus forces) are evaluated using the values of the electrostatic potential (derived from the solution of the Poisson-Boltzmann equation on a three-dimensional grid) generated by one molecule at positions of effective charges of the second molecule. The electrostatic potential grids and effective charges are precomputed so when multiple molecules are simulated, the electrostatic potential grids need to be translated and reorientated at each simulation step along with translating and rotating molecules. This approach ignores the dielectric polarization effects that occur when two molecules come close to each other. These effects may be particularly important in the crowded systems, where intermolecular distances are comparable with the sizes of molecules. Polarization can be accounted for by using an analytical formula proposed by Gabdoulline and Wade [44]. The performance of the effective charges approach is worsened for highly charged molecules in cases where the nonlinear form of the Poisson-Boltzmann equation should be used to evaluate electrostatic potentials. The Debye-Hückel approximation is not valid in these cases, especially in the vicinity of the electrostatic field sources [45].

### Lennard-Jones potentials

Non-specific interactions between molecules can be modeled with the Lennard-Jones type functions that are commonly used in molecular dynamics simulations to compute the van der Waals interactions between atoms; at large intermolecular separations molecules attract each other, while at small separations the interactions are repulsive and molecules are impenetrable. The Lennard-Jones functional form and parameters for BD simulations with coarse-grained spherical models [35,37] can be, for instance, taken from the ones derived for large colloidal spheres [46,47].

In BD simulations employing all-atom models, the forces acting between the surface atoms of molecules can be evaluated using standard (6/12) Lennard-Jones potentials:

(7) $$V\left(r_{ij}\right)=4\epsilon_{LJ}\left({\left(\frac{\sigma_{LJ}}{r_{ij}}\right)}^{12}-{\left(\frac{\sigma_{LJ}}{r_{ij}}\right)}^{6}\right)$$

However, the well depth ϵ*_LJ _*needs to be treated as a free parameter and adjusted to reproduce experimental data [42].

### Hydrophobic effects

Nonpolar (hydrophobic) interactions can be included in atomistic BD simulations by assuming their proportionality to the amount of the solvent accessible surface area (SASA) that gets buried when molecules come close to each other. However, computing of SASA can be slow and to make SASA-based approaches efficient, approximations are needed. Examples of how to incorporate the hydrophobic effects based on SASA in BD simulations can be found in the work of Elcock and McCammon [48] or Gabdoulline and Wade [49].

While SASA-based models are commonly used to evaluate nonpolar interactions, it has been shown that including the solvent accessible volume (SAV) terms may be essential to accurately model the nonpolar solvation, especially at atomic-length scales [50]. Accurate treatment of nonpolar energies and forces is crucial for understanding any biomolecular process that involves changes in solvent accessibility. The described inability of SASA-only models [50] to reproduce and predict solvation energies and forces indicates that there is a need to include a more complete theory of nonpolar solvation in atomistic BD simulations.

It was also proposed that hydrophobic interactions can be accounted for by modifying the van der Waals interactions and assigning more favorable interaction energies to nonpolar surface atoms than to polar ones [42]. With such an approach hydrophobic interactions can be evaluated more rapidly, however its correctness is questioned (see the discussion in [42]).

### Hydrodynamic interactions

In BD simulations water molecules are not treated explicitly but the influence of solvent on the diffusion of suspended molecules can be included by proper treatment of hydrodynamic interactions. However, even though their importance is widely recognized [32,37,51], accounting for HI is no simple matter due to their long-range and many-body character [52] resulting in high computational cost. Therefore, one is often tempted to neglect HI and, consequently, the correlations of motions of molecules.

In the Ermak-McCammon algortihm [23] (Equation 5), hydrodynamic interactions between spherical particles can be included by using the Oseen [53], Rotne-Prager [33] or Rotne-Prager-Yamakawa [34] forms of diffusion tensors (without HI the diffusion tensor is represented by a diagonal matrix). At each step of a BD simulation, the diffusion tensor of the whole system is factorized using the Cholesky decomposition [23] to obtain hydrodynamically correlated displacements of particles. The Cholesky decomposition scales as N^3 ^(with N being the number of particles), while evaluating direct intermolecular interactions, that are assumed pairwise, scales as N^2^. It is possible to lower the cost of evaluating HI using the Chebyshev approximation proposed by Fixman [54], but the HI-related computational overhead is still considerable (N^2.5^). Recently, Geyer and Winter proposed a faster algorithm that scales as N^2 ^and is based on a truncated expansion of the hydrodynamic multi-particle correlations as two-body contributions [55].

An additional computational cost arises when HI are evaluated in finite simulation boxes containing molecules. This cost is due to the fact that for evaluating diffusion tensors Ewald summation has to be used [56,57]. While the direct intermolecular interactions in such periodic systems can be computed using less expensive cutoff -based schemes, applying these schemes to evaluate HI leads to diffusion tensors that are not positive definite (a condition that has to be met to use diffusion tensors as covariance matrices [57].)

The treatment of hydrodynamics within the framework of the Oseen, Rotne-Prager or Rotne-Prager-Yamakwa tensors is appropriate for very dilute systems capturing only the far-field, two-body hydrodynamic effects. However, in crowded biological systems, the many-body HI and lubrication forces (resulting from the thin layer of solvent that separates the nearly touching particles) may significantly influence diffusion. One crude way to include the many-body HI in BD simulations is to use mean-field approaches [58-60] that are based on scaling the diffusion coefficients of particles according to local volume fractions, as for example in the work of Sun and Weinstein [61].

The most advanced approach to treating HI, used recently in BD simulations of a biological system [37], was developed by Brady and Bossis [17]. Their Stokesian dynamics (SD) method includes both far-field and near-field many-body contributions to Brownian forces acting on particles. Moreover, particular implementations of SD enable one to efficiently simulate long-time dynamics of large multicomponent systems. For example, the accelerated SD method, described in [62] scales as N^1.25^log(N).

Unfortunately, modeling of HI in BD simulations is currently limited to systems composed of coarse-grained particles (either spherical molecules or composed of a small number of spherical units - pseudo-atoms). While such models perform well for colloids, a question yet unanswered is whether they are sufficient for crowded, heterogeneous biological systems.

### Applications to diffusion in crowded environments

Typically, the setup of BD simulations aimed at investigating diffusion in crowded solutions is the following. A number of molecules (usually 10^2 ^- 10^3^), described either at the atomistic level or with coarse-grained models, is contained inside a primary simulation box. Periodic boundaries are applied to reduce the edge effects and the influence of the finite size of the system. BD trajectories of all molecules are generated with the algorithm of choice. The BD simulation time scale should be long enough so the relative displacements in the system be at least comparable with the dimensions of molecules; the simulation time scales range from microseconds to milliseconds, while typical time steps are about a few picoseconds. When one considers the number of simulated molecules, the need to describe them in as detailed way as possible, and the complexity of interactions calculated in a single simulation step, it becomes clear that BD simulations of crowded systems require a lot of computational resources.

Below we present a few recent (except for one) examples of BD simulations of macromolecular diffusion in crowded media. We describe the studies that best illustrate the potential and shortcomings of the BD technique applied to in vivo-like systems and the ones that introduce a significant element of novelty. Some of these computational studies relate to experiments - experimental data were either used for parameterization or for direct comparison with simulations.

### Beginnings

One of the first studies that used the BD technique to investigate macromolecular diffusion in congested media was that of Dwyer and Bloomfield [63] in 1993. They performed BD simulations of probe and self-diffusion of concentrated solutions containing short DNA polymers (modeled as wormlike chains, i.e. strings of spherical beads) and the bovine serum albumin (BSA) protein modeled as a sphere. Even though their DNA model lacked the detailed all-atom description, it reproduced the overall translational and rotational behavior of linear DNA molecules [64]. The authors used an algorithm similar to that of Ermak-McCammon [23], however, they neglected HI, because the pairwise approaches are inadequate for concentrated solutions. They also used the Debye-Hückel approach to model long-range electrostatics and the repulsive part of the Lennard-Jones potential to ensure volume exclusion. This model, while very simple, turned out to be realistic enough to simulate diffusion in the DNA/BSA system and accurately reproduce experimental diffusion coefficients at 0.1 M ionic strength for the self-diffusion of BSA and the probe diffusion of BSA in DNA (both over a wide range of BSA/DNA concentrations).

Simulations of the diffusion of BSA in the DNA solution conducted at low ionic strength of 0.01 M gave worse agreement with experiments which the authors attributed to the lack of HI and limitations of the applied electrostatic model. The work of Dwyer and Bloomfield is significant because it shows that even very simple modeling approaches can accurately predict the behavior of rather complex systems.

We note that the models similar to the one of Dwyer and Bloomfield are still used, for example, in computational studies of facilitated diffusion of proteins on DNA. The description of facilitated diffusion phenomenon and models of non-specific protein-DNA interactions can be found in references [65,66].

### Protein-protein association in crowded environments

Most biological reactions require some sort of diffusion-mediated encounter. Here we describe BD applications that directly investigate the influence of crowded environments on diffusional encounter and association of molecules.

Sun and Weinstein [61] simulated systems of spherical particles, mimicking globular proteins. Particles were modeled as hard spheres; the authors used the elastic collision method to effectively deal with particle overlaps [67]. Electrostatic interactions were introduced using the Debye-Hückel approach. A notable fact is that the authors attempted to model also many-body HI. They used the translational part of the Ermak-McCammon algorithm with the diagonal form of the diffusion tensor. However, they applied an average hydrodynamic correction and scaled the diagonal terms of the diffusion tensor (i.e., the single particle diffusion coefficients) with functions that depend on the instantaneous local volume fraction of each particle [58-61]. The authors investigated how the size of background molecules, volume fraction, electrostatic interactions, and HI influence the diffusion of a tracer molecule. Their study also quantitatively described the dependence of diffusion-limited reaction rates on crowding in model systems. Using a very approximate approach, they showed a significant effect of HI on diffusion and association rates. While this review focuses on simulations of multimolecular systems, the importance of intermolecular HI in modeling the association kinetics in a two-molecular case was also described by Frembgen-Kesner and Elcock [32]. A number of simplifications was used in the described work of Sun and Weinstein [61]. Simulated systems were rather homogeneous in comparison with in vivo environments. Molecules were treated as spheres thus their association was non-specific (the model of particles with uniformly reactive surfaces was used). Anisotropy of particles, which may play a role upon association, and specific intermolecular interactions were neglected. However, their work is important as it showed the necessity to include HI in BD simulations of crowded systems.

One of the conclusions of the work of Sun and Weinstein [61] was that crowding slows down the non-specific association of molecules. The two more recent BD studies focused on the influence of crowders on specific interactions in protein-protein association and considered the fact that partner proteins need to be properly oriented to associate. Wieczorek and Zielenkiewicz [68] used BD to simulate the diffusional encounter of HEL and HyHEL-5 proteins in solutions of hard-sphere crowders. Both proteins were modeled with atomistic details, however, only the excluded volume interactions were evaluated; simulation steps leading to overlaps were rejected and repeated with different random displacements, so no systematic forces were explicitly applied. The crowders and proteins were modeled as rigid bodies with isotropic diffusion tensors (both HEL and HyHEL-5 were represented as spheres with equivalent hydrodynamic radii) and propagated with the algorithm described in [25]. Hydrodynamic interactions were neglected. The authors concluded that if the definition of an encounter complex accounts for the orientation of associating molecules, crowding can accelerate the association rate.

Similar observations were made by Kim and Yethiraj [69]. Using spherical protein models with non-uniformly reactive surfaces, they showed that association of model molecules can be either slowed down or accelerated, depending on the applied reaction criteria. These authors recently used BD to investigate the effects of crowding on the association at model membranes [70]. Both studies of Kim and Yethiraj [69,70] focus on how the excluded volume effects influence the reaction rates.

In general, the above simulation results are in agreement with purely theoretical predictions [3]. However, they lack a close connection to experiments and the ability to reproduce heterogeneous in vivo conditions.

### Modeling bacterial cytoplasm

In the recent work of Elcock's [43] and Skolnick's [37] groups BD was applied to investigate the diffusion in cytosol-like systems with the Escherichia coli cytoplasm as a model system. The cytoplasm model of McGuffee and Elcock [43] contains 50 different types of the most abundant proteins and nucleic acids present in the E. coli cytoplasm. Additionally, a few molecules of the Green Fluorescent Protein (GFP) were included, for the sake of comparison with experimental data. All molecules were represented with atomistic details. The algorithm of rigid bodies was applied in BD but all molecules were treated as isotropic, so the average rotational and translational diffusion coefficients were used instead of 6×6 diffusion tensors [25].

Intermolecular interactions included electrostatics, modeled using the effective charges approach of Gabadoulline and Wade [44], and combined van der Waals and hydrophobic interactions described with the Lennard-Jones potential [42], with the well depth treated as an adjustable parameter. Hydrodynamic interactions were neglected. The authors investigated how different energy models affect the translational diffusion coefficient of GFP. When only steric interactions (modeled with the repulsive part of the 6/12 Lennard-Jones potential) were considered, the BD simulated translational diffusion coefficient of GFP was 3 to 6 times higher than the coefficient estimated experimentally. Even including electrostatics did not significantly reduce the discrepancy between the experimental and computed values. Nevertheless, the authors obtained a close agreement with experiments after adding the short-range Lennard-Jones attraction and tuning its well-depth. The observation that purely steric interactions are not able to account for the reduction in proteins' mobilities in vivo concur with previous computational studies [71].

While adjusting the short-range attractive interactions led to reproducing the in vivo GFP diffusion coefficient, it has been later pointed out [37] that the magnitude of attractive interactions can be tuned to set the diffusion coefficient to any chosen value. McGuffee and Elcock investigated also the character of translational diffusion[4] in cytoplasm with different interaction energy models. When only the steric interactions were considered, for the three chosen proteins they observed normal diffusion at short times (ps), subdiffusion at moderate time scales (ns) and normal diffusion at long times (*μ*s). When electrostatic and short-range attractions were included, sub-diffusive behavior was noticed even for short observation times.

The approach to model diffusion in the E. coli cytoplasm taken by Ando and Skolnick [37] is different than the one described above. These authors investigated the effect of macromolecular shapes on the diffusion in crowded, heterogeneous environment, the role of HI, and the interplay between hydrodynamic and non-specific attractive interactions. Their cytoplasm model consisted of 15 different types of molecules, each described with a set of spherical subunits positioned at the C_α _protein atoms and the P, C4', N1, and N9 atoms of nucleic acids. The authors dealt with the anisotropy of macromolecular shapes using the rigid body BD algorithm [26] that describes molecules with precomputed, 6×6 diffusion tensors. Notably, this is the first BD study of a multimolecular system that takes into account the anisotropy of diffusion. To investigate the role of HI they also simulated the system of equivalent spheres corresponding to the cytoplasm model (all molecules in the system were represented as spherical particles and assigned hydrodynamic radii based on translational diffusion coefficients computed from their atomic structures [27]) with the far-field many-body HI interactions and lubrication forces treated with the method of Banchio and Brady [62]. Soft-sphere harmonic potential was used to model repulsive interactions between particles. Attractive interactions were described by the Lennard-Jones potential function with a correction factor accounting for the roughness of molecular surfaces. Electrostatic interactions were modeled using the DLVO model.

Based on simulations of molecular-shaped and equivalent sphere systems, the authors showed that the effects of macromolecular shapes on long-time translational diffusion are rather small in the studied concentration range of 250 - 350 $\frac{mg}{ml}$. This is an important result that justifies the use of isotropic models to study translational diffusion in the crowded systems. The influence of macromolecular shapes on rotational dynamics, which is an interesting issue as well, was not investigated. Their BD simulations with only steric repulsion between particles also resulted in overestimated translational diffusion coefficients. However, when HI were switched on, the computed long-time translational diffusion coefficient of GFP was in good agreement with experimental value; this is another important result because this model did not include any tunable parameters. The authors concluded that the macromolecular motions in vivo are likely to be dominated by steric interactions and hydrodynamics. Additionally, they observed that when non-specific attractive interactions between particles (either molecular-shaped or spherical) are included, the reduction of translational diffusion coefficients depends much more on molecular sizes in comparison with the simulations where HI were enabled.

Both studies described above [37,43] are state of the art applications of BD in the field of macromolecular diffusion in biological systems, as they both take into account the heterogeneity of the environment and intermolecular interactions beyond simple excluded volume models. Both are also closely connected to experimental data.

## Concluding remarks

This short review focuses on the current status of the particle-based Brownian dynamics technique and its applicability to model diffusive phenomena in biological environments. We described a few BD studies of three-dimensional macromolecular diffusion in aqueous cellular compartments under molecular crowding conditions. However, there are various other BD applications, not presented here, that consider diffusive phenomena in cellular architectures, such as the cytoskeleton [72] or membranes [73].

We presented different modeling approaches, from simple excluded-volume models to most sophisticated atomic level descriptions of the in vivo-like systems. The complexity of intra and extracellular environments, their dynamics, and the number of different interactions that influence the diffusive behavior of macromolecules, make computational studies of crowding extremely difficult. While it is desirable to construct models that treat biological systems at atomic resolutions and include sophisticated interactions [42,43], the recent model of the E. coli cytoplasm [37] shows that less elaborate, coarse-grained approaches should not be disregarded, at least as far as diffusive transport is concerned.

Obviously, current BD algorithms need development. These include accurate and computationally efficient models of direct, both specific and non-specific, intermolecular interactions as well as HI models that up to date have been largely neglected in the simulations of biological systems.

BD simulations should be (at least ideally) able to reproduce and predict the in vivo dynamics. However, the computational cost of sophisticated BD simulations may be too high, even with the growth of computing power and available modern technologies. Thus, the long-term goal should be to systematically develop efficient algorithms interfacing different modeling approaches - from atomistic to coarse-grained models of macromolecules, mesoscopic models of biological environments, and appropriate boundary conditions for different cell compartments. New approaches and models should be verified experimentally to establish their quality and predictive power.

## Competing interests

The authors declare that they have no competing interests.

## Authors' contributions

MD studied literature and designed manuscript content. MD and JT wrote the paper.
